# Ultrafine-Grained Structure of Fe-Ni-C Austenitic Alloy Formed by Phase Hardening

**DOI:** 10.1186/s11671-016-1297-9

**Published:** 2016-02-09

**Authors:** Vitalij Danilchenko

**Affiliations:** G.V.Kurdyumov Institute of Metal Physics NAS of Ukraine, Vernadsky blvd. 36, Kiev, 03680 Ukraine

**Keywords:** Iron–nickel alloys, Nanograin, Martensitic transformation, Nanofragmentation, Phase hardening

## Abstract

The X-ray and magnetometry methods were used to study α–γ transformation mechanisms on heating quenched Fe–22.7 wt.% Ni–0.58 wt.% С alloy. Variation of heating rate within 0.03–80 K/min allowed one to switch from diffusive to non-diffusive mechanism of the α–γ transformation. Heating up primary austenitic single crystal specimen at a rate of less than 1.0–0.5 K/min has led to formation of aggregate of grains with different orientation and chemical composition in the reverted austenite. Significant fraction of these grains was determined to have sizes within nanoscale range.

## Background

The nanofragments with low-angle sub-boundaries were found to appear in reverted austenite after fast heating in the temperature interval of the reverse α–γ martensitic transformation [1]. Accordingly, slow heating in this temperature range results in introduction of crystallographic orientations of initial austenite in accordance with orientation relationship between crystal lattices of austenite and martensite (the nanograin refining) [2].

It was determined by the Mossbauer method that aggregate of 30–50 nm thick plate-like crystals with different Ni concentrations was formed in Fe–32 wt.% Ni during slow heating due to redistribution of Ni at interphase boundary between the reverted γ-phase and residual martensite [3]. This process caused formation of concentration inhomogeneity. During the first half of heating through temperature range of the α–γ transformation the dispersed Ni-rich γ-phase with up to 50 wt.% of Ni was observed. The first mention about ability of Ni to be redistributed in the process of the reverse α–γ transformation was in the work [4].

The volume fraction of nanoscale crystals is dependent on complex relationships of different factors—chemical composition of metastable alloys, rate of cooling and following heating in the course of the direct γ–α, and the reverse α–γ transformations, the number of the repeated γ–α–γ transformations (level of the phase hardening) [1, 2, 4–6]. Complexity of these processes calls for additional experimental investigations into formation of grain structure in reverted austenite upon cyclic martensitic transformations. This work deals with the study of heating rate effect on the structural changes during the reverse α–γ transformation in quenched carbon-containing nickel–iron alloy.

## Methods

Fe–22.7 wt.% Ni–0.58 wt.% С alloy was selected to complete the tasks of this study. The alloy was in austenite phase at ambient temperature after quenching from 1270 K into cold water. The direct γ–α transformation occurring on cooling to the liquid nitrogen temperature was followed by the reverse α–γ transformation on heating to 750 ÷ 830 K. Kinetics of martensitic transformations was investigated using differential magnetometer.

Coarse-grained ingot was received by the slow cooling of the melt to 1000 °C with furnace. From 1000 °C, ingot was cooled in cold water. In such ingot, there were the austenitic grains with dimension about 5–10 mm. From the biggest grains, the monocrystal specimens in form of cylinder with diameter of 0.8 mm for the X-ray investigation were cut.

Single crystal diffraction measurements with photo registration were performed in RCW-86 М1 rotation chamber using FeK_α_ radiation. The measurement accuracy of the amount of the martensite by X-ray method is 3 % and by magnetometric method—0.5 %.

## Results and Discussion

**Fig. 1:**
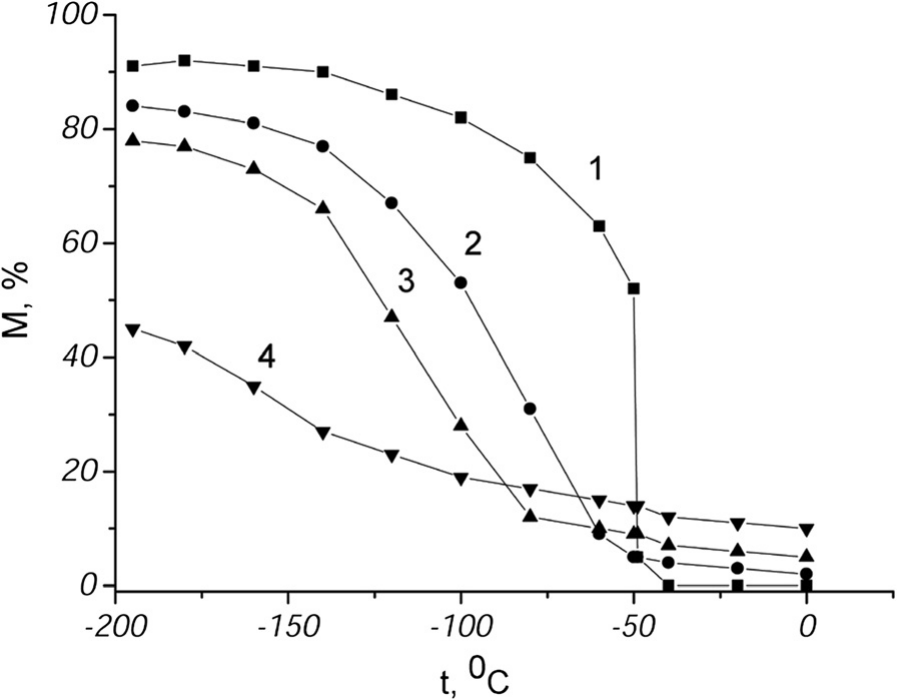
Magnetometric curves of alloy on cooling: *1* the initial state, *2*, *3*, and *4* after the α–γ transformation, realized at a heating rate of 60; 0, 08; 0,05 K/min, respectively

The magnetometry measurements have revealed high degree of correlation between completeness of the direct γ–α transformation and the rate of heating in the interval of the reverse α–γ transformation. The start temperature of the γ–α transformation and the fraction of martensitic phase formed on cooling down to liquid nitrogen temperature were reduced as the heating rate of the direct transformation was decreased in the Fe–22.7 wt.% Ni–0.58 wt.% С alloy (Fig. 1).

Heating of the alloy at a rate higher than 2–3 K/min did not lead to significant change of the kinetics and the completeness of the following direct γ–α transformation. If the heating of the quenched alloy occurs at a rate lower than critical one, the α–γ transformation is accompanied by diffusion processes, which result in gradual decreasing the completeness of the γ–α transformation and enrichment of the reverted austenite with nickel.

The change of heating rate within certain range in the temperature interval of the α–γ transformation allows one to control relationship between diffusive and non-diffusive components of its mechanism. Accordingly, realization of the reverse transformation gives rise to formation of high-dispersive structure of the reverted austenite at nanoscale level. Slow heating up to start temperatures of the reverse transformation caused non-recoverable changes in the original orientation of the austenite lattice as it was in case of rapid heating of quenched alloys to these temperatures. Diffusive character of the reverse transformation led to multiplication of orientations of the reverted austenite. Principally, different mechanism compared to those involved in intensive plastic deformation, evaporation, or gas deposition accounts for nanocrystalline heterophase system formation due to cyclic direct and reverse martensitic transformations. According to this mechanism, the fragmentation factor is related to crystallography of martensitic transformations. Multiplication of orientations associated with the γ–α–γ transformations is accounted for by nucleation of γ-crystal inside every α-crystal, while all 24 martensite variants tend to be realized upon the α–γ transformation [1, 2, 7].

Tuning the heating rate of the quenched carbon-containing nickel-iron alloys within the range of 0.03 ÷ 80 K/min makes it possible to change relationship between diffusive and non-diffusive components of the transformation mechanism.

The X-ray study of the structure of phase-hardened single crystal alloys revealed structural mechanism of the reverse α–γ transformation [2]. Heating the specimen in the temperature interval of the α–γ transformation at a rate higher than 0.5 K/min leads to formation of reverted austenite with orientation corresponding to non-diffusive character of the reverse transition [1, 3]. When the heating rate was reduced down to 0.05 K/min, reflections from the γ-phase caused by significant number of new grains were observed. New grains had different orientations in relation to those of the initial austenite. They were located within certain interval around the Bragg angle. Thus, interval for the (311)_γ_ peak was between 111.58° and 112.59°. Single reflections located in certain interval of the Bragg angle transformed in practically continuous diffraction line as a regime of X-ray measurements provided oscillations of specimen within the angular interval of ±12°. The reverted γ-phase peaks gained all values located in between the minimum and the maximum Bragg angle indicating continuous build-up of nickel concentration in the γ-phase.

**Fig. 2:**
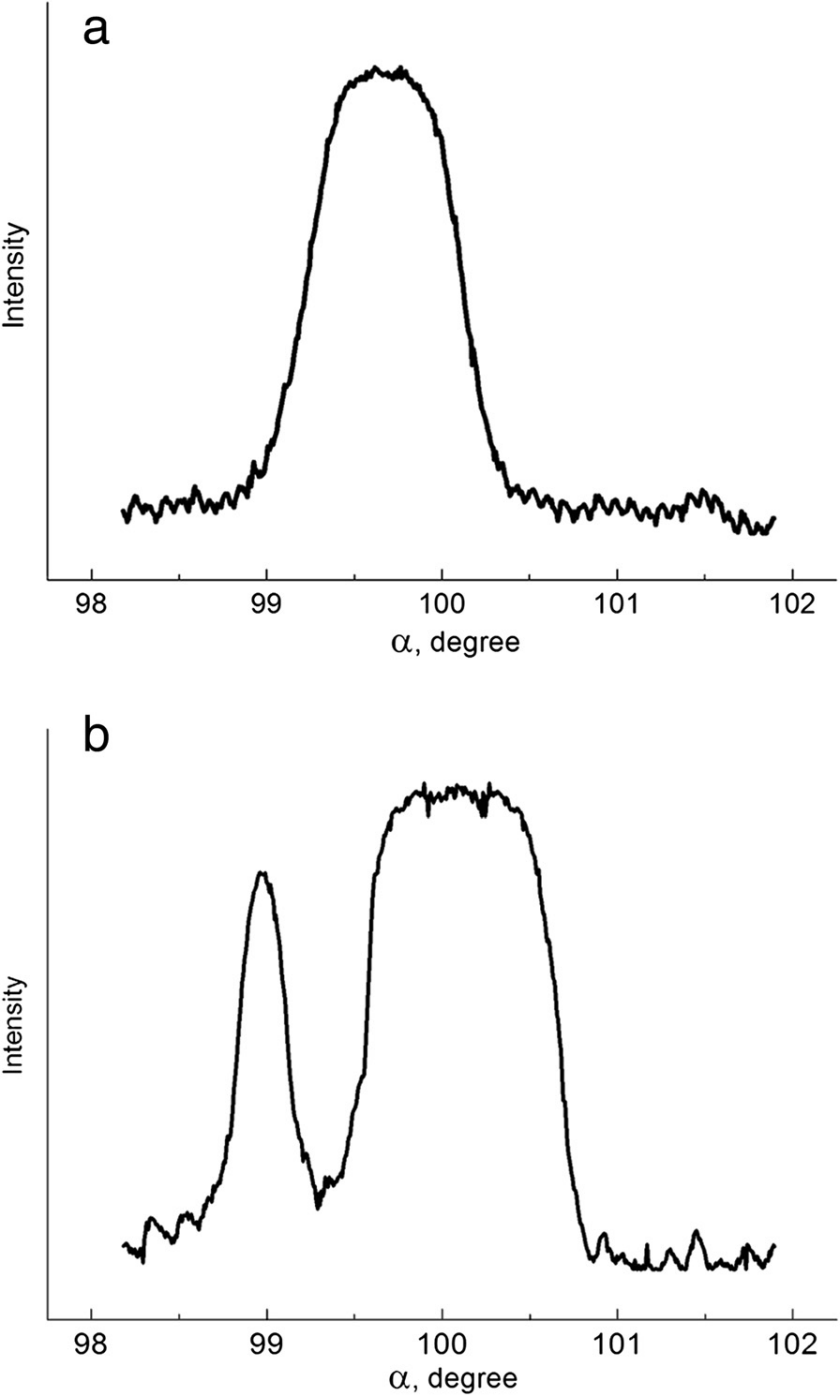
Microphotometric curves of the (220)_γ_ reflections from reverted austenite formed during non-diffusive (**a**) and diffusive (**b**) α–γ transformations

**Fig. 3:**
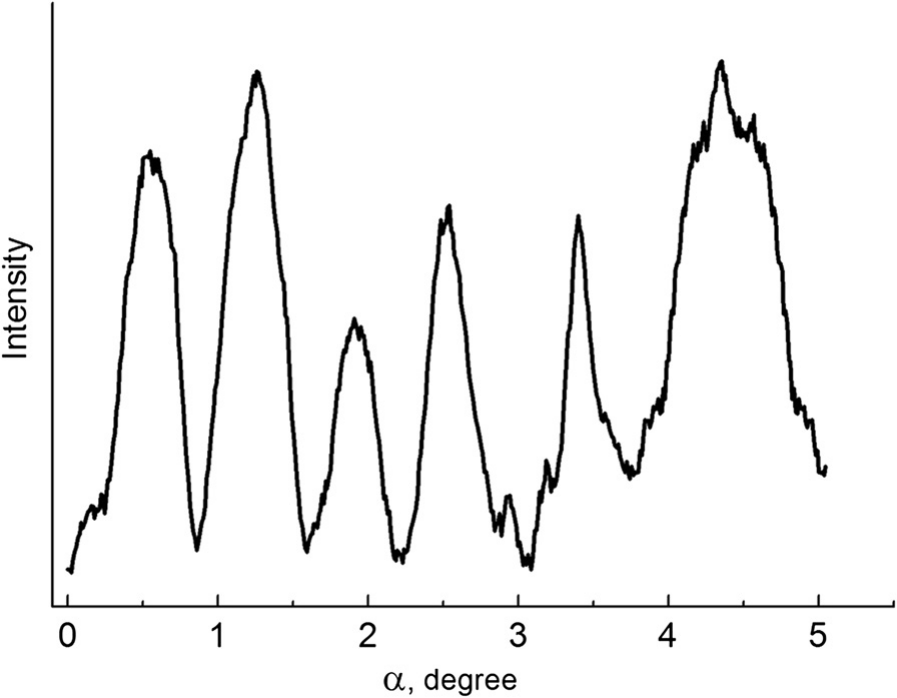
Microphotometric curve along the azimuthal direction of the (220)_γ_ reflection from reverted austenite formed during diffusive α–γ transformation

Microphotometric curves of the reverted γ-phase reflexes have shown considerably different peak broadening for diffusive and non-diffusive α–γ transformation. In case of diffusive α–γ transition, higher broadening was caused by number of reflections from grains with different nickel content (Fig. 2). The half-width of certain reflexes appearing at diffusive α–γ transformation was found to be much smaller than at non-diffusive α–γ transformation. It occurred due to relaxation processes minimizing the influence of internal stresses on reflection broadening upon slow cooling in the temperature interval of the α–γ transition. Occurrence of the additional separate reflections in azimuthal direction of the initial reflection (220)_γ_ testified on the formation of new grains during the diffusive α–γ transformation (Fig. 3).

Intensity of the reflexes varied over an order of magnitude and more that implied the number of grains of different orientations were noticeably different. The most intensive peaks corresponded orientations close to orientations of the austenite twinned with respect to the initial one. The formation of twinned austenite can be expected in regions of the reverted γ-phase where relaxation processes occur to their full extent. Some number of reflections similar to spot ones and practically without azimuthal blurring as well as the reflections with azimuthal blurring measured several degrees were observed. It indicated inhomogeneity of grain structure of the reverted austenite.

Certain peaks were accompanied by diffusion effects (tails) characteristic of thin plate-like dispersed grains of nanoscale size. Appearance of doublet reflections corresponding to almost the same orientations but different Bragg angles can be attributed to concentration inhomogeneity of nickel in grains.

## Conclusions

It has been shown that the reverse α–γ transformation by the diffusion mechanism in the Fe–22.7 wt.% Ni–0.58 wt.% С alloy gives rise to the formation of complex grain structure of reverted austenite. The diffraction pattern of the reverted austenite has not demonstrated signs of internal stress. The half-width of diffraction peaks of the reverted austenite was significantly smaller than that of the initial austenite. The reverted austenite grains formed in the course of α–γ transformation displayed different orientations and sizes including nanoscale ones. They also possessed significant concentration inhomogeneity characterized by continuous change of nickel concentration in the whole range of its values.

## References

[1] Kabanova IG, Sagaradze VV, Kataeva NV, Danilchenko VE (2011). Detection of the ε phase and the Headley-Brooks orientation relationships upon α → γ transformation in the Fe-32 % Ni alloy. Phys Met Metallogr.

[2] Danilchenko V, Sagaradze V, Dzevin I (2013). Effect of multiple martensitic transformations on structure of Fe-Ni alloys. J Mater Sci Technol.

[3] Lysak LI, Danilchenko VE, Polischuk UM, Ustinov AI (1975). Twinning on {011} <011 > system of martensite of the manganese steel. Reports of USSR Academy of Sciences.

[4] Krauss G, Cohen J, Cohen M (1962). Strengthening and annealing of austenite formal by the reverse martensitic transformation. Trans AIME.

[5] Kessler H, Pitsch W (1967). On the nature of the martensite to austenite reverse transformation. Acta Met.

[6] Danilchenko VE, Sagaradze VV, L’Heritier P (2003). Martensite crystal structure of nickel steel at cryogenic temperatures. Mat Sci Eng.

[7] Lysak LI, Nickolin BI (1975). Physical basis of heat treatment of steel.

